# Supplementary material for: Yiai Fuzheng decoction inhibits triple-negative breast cancer by remodeling the immune microenvironment
Source: Front Immunol. 2025 Sep 30;16:1615631. doi: 10.3389/fimmu.2025.1615631 (PMC12518410; doi:10.3389/fimmu.2025.1615631)
Supplement: Supplementary file 2 [file DataSheet1.zip › Supplementary File 2/Treatment/C_vs_H/07_Pathway_Analysis/Pathway_Analysis/Sig_Pathways_Name/Glycerolipid metabolism.pdf]

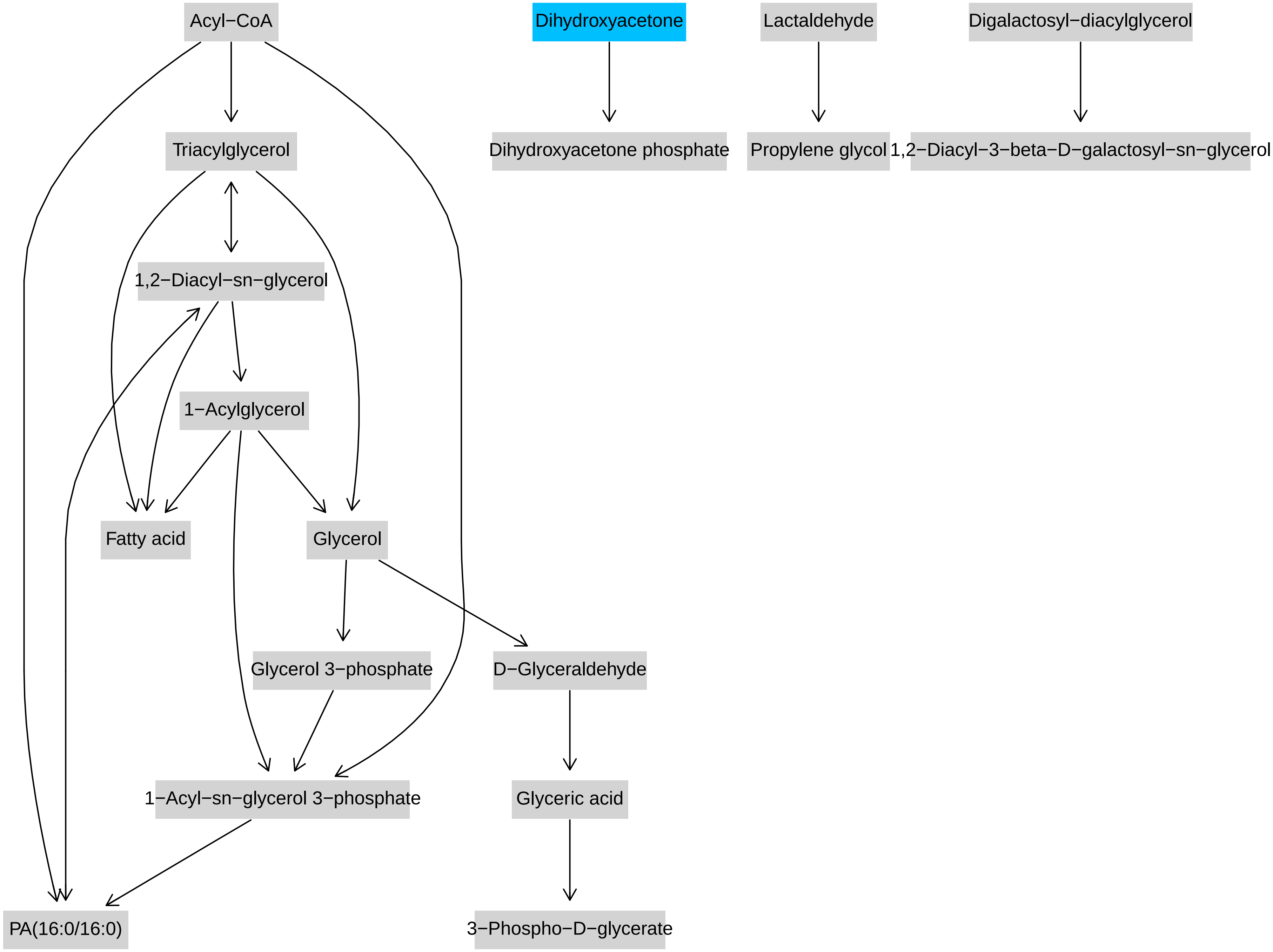

Acyl−CoA
Dihydroxyacetone
Lactaldehyde
Digalactosyl−diacylglycerol
Triacylglycerol
Dihydroxyacetone phosphate
Propylene glycol
1,2−Diacyl−3−beta−D−galactosyl−sn−glycerol
1,2−Diacyl−sn−glycerol
1−Acylglycerol
Fatty acid
Glycerol
Glycerol 3−phosphate
D−Glyceraldehyde
1−Acyl−sn−glycerol 3−phosphate
Glyceric acid
PA(16:0/16:0)
3−Phospho−D−glycerate
